# Association of body mass index and sexual dysfunction among married women in Makkah City, Saudi Arabia

**DOI:** 10.3389/fgwh.2025.1616496

**Published:** 2025-07-31

**Authors:** Lujain Safwan Filfilan, Nesrin Kamal Abd El-Fatah

**Affiliations:** ^1^ Postgraduate Program for Preventive Medicine, Public Health Administration, Taif Health Cluster, Ministry of Health, Taif, Saudi Arabia; ^2^ Department of Nutrition, High Institute of Public Health, Alexandria University, Alexandria, Egypt

**Keywords:** female sexual dysfunction, Obesity, BMI, Saudi Arabia, marital satisfaction, reproductive health

## Abstract

**Background:**

Female sexual dysfunction (FSD) is a prevalent yet understudied health concern among married women in Saudi Arabia, where obesity rates among women are alarmingly high. This study aimed to investigate the prevalence of FSD and its association with BMI among married women attending primary health care centers (PHCs) in Makkah, Saudi Arabia.

**Methods:**

A cross-sectional study was conducted among 332 married women aged 18–50 years attending PHCs in Makkah. Participants completed a structured questionnaire assessing sociodemographics, medical history, reproductive health, and sexual function using the validated Arabic Female Sexual Function Index (ArFSFI). Anthropometric measurements were taken to calculate Body Mass Index (BMI). Chi-square tests, logistic regression, and Kruskal–Wallis analyses were used to examine associations between BMI, FSD, and significant contributors.

**Results:**

The prevalence of FSD was 84.6%. Obesity was significantly associated with FSD (OR = 2.86, 95% CI: 1.38–5.30, *p* = .005), particularly affecting lubrication (*p* = .002) and orgasm (*p* = .014). Key correlates of FSD included partner-related factors, such as the husband's sexual dysfunction (40.9% vs. 5.9%, *p* < .001), and weight-related comments (33.8% vs. 15.7%, *p* = .010). Psychological distress, particularly higher levels of anxiety (32.0% vs. 11.8%, *p* = .003) and depression (44.5% vs. 7.8%, *p* < .001), was also significantly associated with FSD. Reproductive factors, such as irregular menstruation (*p* = .001), and reproductive surgeries (*p* = .003) were significantly associated with FSD.

**Conclusion:**

This study highlights a high burden of FSD among Saudi women with obesity, strongly associated with obesity, mediated by psychological and partner-related factors, as well as reproductive factors. Integrated interventions addressing weight management, mental health, and couples' counseling are urgently needed. Cultural sensitivity in sexual health education and clinical practice is essential to reduce stigma and improve care access.

## 1 Introduction

Sexuality is a fundamental aspect of health and well-being, significantly influencing quality of life and interpersonal relationships (1). Female Sexual Dysfunction (FSD) is a multifaceted condition characterized by disturbances in sexual desire, arousal, orgasm, or pain, leading to personal distress (1, 2). However, FSD remains understudied and underdiagnosed, particularly in conservative societies such as Saudi Arabia. Epidemiological data indicate that FSD affects 25–63% of women globally, with higher rates observed in regions where cultural and social factors may impede open discussion or seeking treatment (3, 4). In Saudi Arabia, limited studies have explored FSD, reporting a prevalence rate as high as 68% among married women, highlighting a critical public health gap (5, 6).

Obesity is another pressing health issue in Saudi Arabia, with 33.5% of women classified as obese (7, 8). Obesity is linked to chronic conditions such as diabetes and cardiovascular disease, but its association with FSD is less clear (9). While some studies suggest that higher Body Mass Index (BMI) correlates with sexual dysfunction due to psychological (e.g., poor body image) and physiological (e.g., hormonal imbalances) factors (10, 11), others report no significant relationship (12). This inconsistency underscores the need for further research, particularly in populations where obesity rates are high and cultural norms may exacerbate body image concerns (13).

The interplay between BMI and FSD is complex and likely mediated by variables such as mental health, partner dynamics, and sociodemographic factors (14). For instance, depression and anxiety (common among obese individuals) are known contributors to FSD (15). Additionally, partner health, marital satisfaction, and frequency of sexual activity may modify this relationship (16). Previous studies in Saudi Arabia have focused narrowly on FSD prevalence or obesity alone, neglecting their potential interaction (6, 8, 17). Moreover, confounding factors like reproductive stage, medication use (e.g., selective serotonin reuptake inhibitors), and lifestyle habits (e.g., physical inactivity) are often overlooked (18). Thus, the lack of local data and conflicting global evidence highlights the need for further studies in Saudi Arabia.

To study this gap in the literature, the aim of this study was to determine the prevalence and predictors of FSD among Saudi married women, and to investigate FSD association with BMI. The findings provided evidence to guide interventions targeting sexual health in obese women, potentially improving quality of life and reducing marital disputes. The findings could also contribute to the limited literature on FSD in Saudi Arabia and inform culturally sensitive health policies. Understanding how obesity influences sexual function can help clinicians address FSD holistically, integrating weight management and psychological support into care plans.

## 2 Methods

### 2.1 Study design and population

A cross-sectional study was conducted on married women attending PHCs in Makkah, Saudi Arabia, between November 2024 to January 2025. The study employed an analytical design to assess the association between BMI and FSD. Makkah was selected due to its diverse population and high prevalence of obesity, as reported in national health surveys. Married Saudi women aged 18–50 years who were sexually active in the preceding four weeks were included, while non-Saudi or unmarried women, and those with psychiatric disorders (as self-reported) were excluded. Using Epi Info 7, the minimum required sample was 329 participants based on a previously reported FSD prevalence of 68.9% (19), with a 95% confidence level and 5% margin of error. A total of 18 PHCs serve the city, of which three were randomly selected using a simple randomization technique to ensure representativeness. Participants were recruited consecutively during their routine visits until the required sample size was achieved. It is noteworthy to mention that the response rate was almost 94%.

### 2.2 Data collection tools and procedures

Data were collected using a structured, interviewer-administered questionnaire divided into several sections to comprehensively assess sociodemographic, medical, and sexual health factors.

#### 2.2.1 Socio-demographic and reproductive characteristics

This section gathered information on age, education level, employment status, and monthly income. Additionally, participants provided details about Age of menarche, marriage duration, parity, and mode of delivery (vaginal or cesarean). Data on contraceptive use, history of gynecological surgeries, and menopausal status were also collected.

#### 2.2.2 Medical and lifestyle history

Lifestyle factors, such as smoking, physical activity, and sleep duration, were assessed. Further assessments included history of chronic illnesses and medications known to affect sexual health, including selective serotonin reuptake inhibitors (SSRIs) and hormonal therapy.

#### 2.2.3 Assessment of female sexual function

Sexual function was evaluated using the Arabic version of the Female Sexual Function Index (ArFSFI), a validated 19-item tool (20). The ArFSFI assesses six domains over the past four weeks: desire, arousal, lubrication, orgasm, satisfaction, and pain during intercourse. Each domain is scored on a scale of 0–5, with a total score of ≤28.1 indicating the presence of FSD (21).

#### 2.2.4 Psychological assessment

The PHQ-4 was administered to evaluate mental health status. It is a short 4-item questionnaire designed to measuring anxiety and depression symptoms experienced over the past two weeks (22). consists of two subscales, anxiety and depression, each containing two items. Each item is rated on a 4-point Likert scale ranging from 0 (not at all) to 3 (nearly every day). PHQ4 scores are categorized into levels of psychological distress severity. A score of 0 to 2 indicates the absence of psychological distress. A score from 3-5, 6-8 and 9-12, indicate mild, moderate and severe psychological distress.

#### 2.2.5 Anthropometric measurements

Trained personnel measured participants’ weight and height using standardized protocols. Weight was measured in light clothing using a calibrated digital scale (to the nearest 0.1 kg), while height was measured without shoes using a stadiometer (to the nearest 0.1 cm). BMI was calculated as weight in kilograms divided by height in meters squared (kg/m^2^) and classified according to World Health Organization (WHO) criteria: underweight (<18.5 kg/m^2^), normal weight (18.5–24.9 kg/m^2^), overweight (25–29.9), and obesity ≥30 kg/m^2^.

### 2.3 Pilot testing and data quality control

A pilot study involving 20 participants was conducted to test the clarity and feasibility of the questionnaire. Feedback from the pilot phase led to minor refinements in the wording and flow of questions. Data collection was carried out by trained female interviewers to minimize bias and ensure consistency. Completed questionnaires were reviewed for accuracy and completeness before data entry. The internal consistency of the questionnaire demonstrated good reliability with Cronbach's *α* = 0.85.

### 2.4 Statistical analysis

Data was analyzed using version 27.0 of the IBM SPSS software package (Armonk, NY: IBM Corp), and visualization was performed using Microsoft Excel 365. Quantitative data were reported using minimum, maximum, mean, and standard deviation, whereas qualitative data were described using numbers and percentages (%). The Kolmogorov–Smirnov test was performed to determine the date's normality. The relationship between two qualitative variables was analyzed by Utilizing the Chi-square test. The comparison between more than two quantitative variables with no normal distribution was examined using the Kruskal–Wallis H test. In addition, a univariate logistic regression analysis was utilized to determine the odds ratio. Values less than 0.05 were judged to be statistically significant, and values less than 0.01 were considered to be highly statistically significant.

### 2.5 Ethical considerations

The research received institutional ethical approval from the Research Ethics Committee of Taif Health Affairs, Ministry of Health, Saudi Arabia (IRB: HAP-02-T-067, Number 834). Written informed consent was obtained from all participants after explaining the study objectives, procedures, and their right to withdraw at any time without consequences. Confidentiality was strictly maintained, and all data were anonymized to protect participant privacy.

## 3 Results

A total of 332 married women were involved in the study and classified according to the ArFSFI cutoff value into normal group [*n* = 51, 15.4%] and dysfunction group [*n* = 281, 84.6%]. Table 1 shows sociodemographic characteristics of study participants by FSFI. Significant differences between married women with normal FSFI scores and those with dysfunction were observed for age, income, participants education and Husband's Employment (*p* < 0.05). Significant higher proportions of dysfunction were observed among those younger than 30 and those older than 35, compared to the 31-35 age group. The husband's age was also significantly associated with the wife's FSFI status (*p* = .020). A higher percentage of women experiencing sexual dysfunction had husbands aged 56-65 (17.4%), and over 65 (8.2%). Residence also showed a significant association with FSFI status where 83.6% of the FSFI dysfunction group reported living in urban areas. Women reporting lower income (7501-11000 Rial/month) and lower education were more prevalent in the dysfunction group, while those with a higher income, a university educational degree, and employed husbands, were slightly more represented in the normal group (29.4% and 72.5%, 74.5%) respectively). No statistically significant associations were found between the wife's FSFI status and husband's education level (*p* = .161), wife's employment status (*p* = .247), or living arrangements with the husband (*p* = .153).

**Table 1:** Sociodemographic characteristics of study participants by female sexual function Index Status (*N* = 332).

Characteristics	Total	ArFSFI		*P*
	*N* = 332	Normal (*n* = 51, 15.4%)	Dysfunction (*n* = 281, 84.6%)	
Age				
Less than 18	7 (2.1%)	0 (0.0%)	7 (2.5%)	<.001**
18-25	13 (3.9%)	1 (2.0%)	12 (4.3%)	
26-30	21 (6.3%)	2 (3.9%)	19 (6.8%)	
31-35	83 (25.0%)	23 (45.1%)	60 (21.4%)	
36-40	53 (16.0%)	6 (11.8%)	47 (16.7%)	
41-45	63 (19.0%)	13 (25.5%)	50 (17.8%)	
46-50	66 (19.9%)	0 (0.0%)	66 (23.5%)	
51-55	16 (4.8%)	5 (9.8%)	11 (3.9%)	
More than 55	10 (3.0%)	1 (2.0%)	9 (3.2%)	
Husband's age				
Less than 18	15 (4.5%)	5 (9.8%)	10 (3.6%)	.020*
18-25	14 (4.2%)	2 (3.9%)	12 (4.3%)	
26-35	44 (13.3%)	9 (17.6%)	35 (12.5%)	
36-45	93 (28.0%)	20 (39.2%)	73 (26.0%)	
46-55	91 (27.4%)	12 (23.5%)	79 (28.1%)	
56-65	51 (15.4%)	2 (3.9%)	49 (17.4%)	
More than 65	24 (7.2%)	1 (2.0%)	23 (8.2%)	
Residence				
Urban	286 (86.1%)	51 (100.0%)	235 (83.6%)	.002**
Rural	46 (13.9%)	0 (0.0%)	46 (16.4%)	
Average family income				
Less than 3500	21 (6.3%)	2 (3.9%)	19 (6.8%)	.018*
3501–7500	40 (12.0%)	8 (15.7%)	32 (11.4%)	
7501–11000	75 (22.6%)	5 (9.8%)	70 (24.9%)	
11001–14500	22 (6.6%)	8 (15.7%)	14 (5.0%)	
14501–18000	92 (27.7%)	13 (25.5%)	79 (28.1%)	
More than 18000	82 (24.7%)	15 (29.4%)	67 (23.8%)	
Participant's education				
Illiterate/read &write	8 (2.4%)	0 (0.0%)	8 (2.8%)	<.001**
Primary education	26 (7.8%)	1 (2.0%)	25 (8.9%)	
High school	58 (17.5%)	0 (0.0%)	58 (20.6%)	
University degree	174 (52.4%)	37 (72.5%)	137 (48.8%)	
Postgraduate	66 (19.9%)	13 (25.5%)	53 (18.9%)	
Husband's education				
Illiterate/read &write	9 (2.7%)	1 (2.0%)	8 (2.8%)	.161
Primary education	17 (5.1%)	0 (0.0%)	17 (6.0%)	
High school	84 (25.3%)	9 (17.6%)	75 (26.7%)	
University degree	155 (46.7%)	30 (58.8%)	125 (44.5%)	
Postgraduate	67 (20.2%)	11 (21.6%)	56 (19.9%)	
Wife's employment				
Student	19 (5.7%)	2 (3.9%)	17 (6.0%)	.247
Housewife	114 (34.3%)	20 (39.2%)	94 (33.5%)	
Employed	161 (48.5%)	28 (54.9%)	133 (47.3%)	
Self-employed	9 (2.7%)	1 (2.0%)	8 (2.8%)	
Military employee	10 (3.0%)	0 (0.0%)	10 (3.6%)	
Retired	19 (5.7%)	0 (0.0%)	19 (6.8%)	
Husband's employment				
Student	1 (0.3%)	0 (0.0%)	1 (0.4%)	.010**
Employed	179 (53.9%)	38 (74.5%)	141 (50.2%)	
Self-employed	47 (14.2%)	2 (3.9%)	45 (16.0%)	
Military employee	51 (15.4%)	8 (15.7%)	43 (15.3%)	
Retired	54 (16.3%)	3 (5.9%)	51 (18.1%)	
Living with husband				
No	37 (11.1%)	2 (3.9%)	35 (12.5%)	.153
Husband travels frequently	33 (9.9%)	4 (7.8%)	29 (10.3%)	
Yes	262 (78.9%)	45 (88.2%)	217 (77.2%)	

FSFI, female sexual function index.

Values presented are frequency (n) and column percentage (%). *P*-values were derived from Chi-square tests comparing the distribution of each variable between the FSFI normal and FSFI dysfunction groups.

Bold values indicate statistical significance.

* Significant at *P* ≤ 0.05.

** Significant at *P* ≤ 0.01.

Table 2 presents the reproductive and gynecological characteristics by FSFI. A significantly higher proportion of women in the normal group reported neither pregnancy nor breastfeeding (*n* = 46, 90.2%) and all women who were currently pregnant or breastfeeding (*n* = 42, 14.9% of the dysfunction group) fell into the FSFI dysfunction category (*p* = .004). Women reporting menarche age between 10-12 years were more represented in the normal group (47.1%). In contrast, those who experienced menarche later (15-17 years or over 17 years) were exclusively found in the dysfunction group (15.7% and 2.8%, respectively). Menstrual cycle regularity was significantly different between the groups (*p* = .001), where women experiencing menopause (15.7%) or lactation-related amenorrhea (5.3%) were only present in the dysfunction group. Likewise, the longer duration of menstrual cycle days and Contraceptive use were significantly associated with sexual dysfunction (*p* = .006,.007 and.040 respectively). Women whose delivery was exclusively vaginal were more common in the normal function group (47.1% vs 29.5%, *p* = .014). Finally, reproductive surgeries were significantly more prevalent among women with sexual dysfunction (15.7%) compared to those with normal function (2.0%) (*p* = .008). In contrast, no statistically significant associations were found between FSFI status and age at marriage, marriage duration, being in a polygamous marriage, history of gynecological diseases (*p* = .175), use of hormonal treatments (*p* = .157), overall pregnancy history (*p* = .875), or the number of children (*p* = .189).

**Table 2:** Reproductive and gynecological characteristics of married women primary health care attendees in makkah by female sexual function index status (*N* = 332).

Characteristics	Total	ArFSFI		*P*
	*N* = 332	Normal (*n* = 51, 15.4%)	Dysfunction (*n* = 281, 84.6%)	
Current pregnancy/breastfeeding				
No pregnancy, No breastfeeding	241 (72.6%)	46 (90.2%)	195 (69.4%)	.004*
No pregnancy, breastfeeding	49 (14.8%)	5 (9.8%)	44 (15.7%)	
Yes (pregnant or breastfeeding)	42 (12.7%)	0 (0.0%)	42 (14.9%)	
Age at marriage				
Less than 18	17 (5.1%)	3 (5.9%)	14 (5.0%)	.970
18-21	67 (20.2%)	10 (19.6%)	57 (20.3%)	
22-25	109 (32.8%)	18 (35.3%)	91 (32.4%)	
26-30	86 (25.9%)	12 (23.5%)	74 (26.3%)	
31-35	24 (7.2%)	4 (7.8%)	20 (7.1%)	
36-40	15 (4.5%)	3 (5.9%)	12 (4.3%)	
More than 40	14 (4.2%)	1 (2.0%)	13 (4.6%)	
Marriage duration				
Less than 2	21 (6.3%)	4 (7.8%)	17 (6.0%)	.470
2-4	21 (6.3%)	1 (2.0%)	20 (7.1%)	
4-7	32 (9.6%)	7 (13.7%)	25 (8.9%)	
8-10	56 (16.9%)	8 (15.7%)	48 (17.1%)	
11-15	69 (20.8%)	14 (27.5%)	55 (19.6%)	
16-20	59 (17.8%)	9 (17.6%)	50 (17.8%)	
More than 20	74 (22.3%)	8 (15.7%)	66 (23.5%)	
Polygamous marriage	44 (13.3%)	4 (7.8%)	40 (14.2%)	.216
Gynecological diseases	128 (38.6%)	24 (47.1%)	104 (37.0%)	.175
Age of menarche				
Under 10 years	22 (6.6%)	2 (3.9%)	20 (7.1%)	.011*
10-12 years	117 (35.2%)	24 (47.1%)	93 (33.1%)	
13-15 years	141 (42.5%)	25 (49.0%)	116 (41.3%)	
15-17 years	44 (13.3%)	0 (0.0%)	44 (15.7%)	
Over 17 years	8 (2.4%)	0 (0.0%)	8 (2.8%)	
Menstrual cycle regularity				
Regular	209 (63.0%)	43 (84.3%)	166 (59.1%)	.001**
Irregular	64 (19.3%)	8 (15.7%)	56 (19.9%)	
Menopause	44 (13.3%)	0 (0.0%)	44 (15.7%)	
Lactational amenorrhea	15 (4.5%)	0 (0.0%)	15 (5.3%)	
Menstrual cycle days				
Two days or less	10 (3.0%)	0 (0.0%)	10 (3.6%)	.006**
3-4 days	56 (16.9%)	16 (31.4%)	40 (14.2%)	
5-7 days	218 (65.7%)	32 (62.7%)	186 (66.2%)	
More than 7 days	48 (14.5%)	3 (5.9%)	45 (16.0%)	
Hormonal therapy	39 (11.7%)	3 (5.9%)	36 (12.8%)	.157
Previous pregnancies	314 (94.6%)	48 (94.1%)	266 (94.7%)	.875
Number of children				.195
One	65 (19.6%)	17 (34%)	56 (18.1%)	
Two	87 (26.2%)	10 (19.6%)	77 (27.4%)	
Three	77 (23.2%)	13 (25.5%)	64 (22.8%)	
Four	43 (13.0%)	6 (11.8%)	37 (13.2%)	
More than four	52 (15.7%)	5 (9.8%)	47 (16.7%)	
Previous delivery methods				
Vaginal	107 (32.2%)	24 (47.1%)	83 (29.5%)	.014*
Cesarean	132 (39.8%)	20 (39.2%)	112 (39.9%)	
Both	93 (28.0%)	7 (13.7%)	86 (30.6%)	
Current contraceptive use	161 (48.5%)	18 (35.3%)	143 (50.9%)	.040*
Reproductive surgeries !	45 (13.6%)	1 (2.0%)	44 (15.7%)	.008**

FSFI, female sexual function index.

*P*-values were derived from Chi-square tests.

Bold values indicate statistical significance.

* Significant at *P* ≤ 0.05.

** Significant at *P* ≤ 0.01. **!** surgeries other than Cesarean section.

Table 3 shows the association of FSFI status and lifestyle, medical, and mental histories among the studied females. Significant differences were observed for sleep duration, substance use, whether the husband requested weight loss, marital satisfaction and psychological distress level (*p* < 0.05). Sleeping > 9 h was reported more frequently by women in the dysfunction group (14.6%) than the normal group (3.9%). While all women in the normal function group reported no use of alcohol or drugs, 10.0% of women in the dysfunction group reported using these substances. A considerably larger percentage of women in the dysfunction group reported that their husbands had requested weight loss (33.8%) compared to women in the normal function group (15.7%). Similarly, the prevalence of anxiety and depression were significantly higher among women with sexual dysfunction (32.0% and 44.5% respectively) compared to women with normal sexual function (11.8% and 7.8%). The overall PHQ4 score, was significantly different between the groups (*p* < .001) where women with moderate (26.7%) and severe (7.1%) distress levels were considerably more common in the FSFI dysfunction group compared to the normal group (3.9% and 0.0%, respectively). In contrast, no statistically significant associations were found between FSFI status and the participant's own smoking status (*p* = .071), husband's smoking status (*p* = .178), regular physical activity (*p* = .878), specific weight loss methods attempted (*p* = .852), history of chronic diseases (*p* = 0.077), bariatric surgery (*p* = 0.74), or self-reported body shape satisfaction (*p* = .395).

**Table 3:** Lifestyle, medical, mental health and sexual histories of married women primary health care attendees in makkah by female sexual function Index Status (*N* = 332).

Histories	Total	ArFSFI		*P*
	*N* = 332	Normal (*n* = 51, 15.4%)	Dysfunction (*n* = 281, 84.6%)	
Smoking status (self)				
Non-smoker	274 (82.5%)	40 (78.4%)	234 (83.3%)	0.071
Former smoker	20 (6.0%)	1 (2.0%)	19 (6.8%)	
Smoker	38 (11.4%)	10 (19.6%)	28 (10.0%)	
Smoking status (husband)				
Non-smoker	148 (44.6%)	21 (41.2%)	127 (45.2%)	0.178
Former smoker	48 (14.5%)	4 (7.8%)	44 (15.7%)	
Smoker	136 (41.0%)	26 (51.0%)	110 (39.1%)	
Sleep duration				
≤7 hours	178 (53.6%)	26 (51.0%)	152 (54.1%)	.042*
>7-9 hours	111 (33.4%)	23 (45.1%)	88 (31.3%)	
>9 hours	43 (13.0%)	2 (3.9%)	41 (14.6%)	
Physically activity	127 (38.3%)	20 (39.2%)	107 (38.1%)	0.878
Substance abuse/alcohol	28 (8.4%)	0 (0.0%)	28 (10.0%)	.018*
Weight Loss method attempted				
Never tried	79 (23.8%)	10 (19.6%)	69 (24.6%)	0.852
Using medication	41 (12.3%)	6 (11.8%)	35 (12.5%)	
Bariatric surgery	21 (6.3%)	4 (7.8%)	17 (6.0%)	
Dieting	191 (57.5%)	31 (60.8%)	160 (56.9%)	
Bariatric surgery history	21 (6.3%)	4 (7.8%)	17 (6.0%)	0.74
Body shape satisfaction				0.395
Very dissatisfied	36 (10.8%)	4 (7.8%)	32 (11.4%)	
Dissatisfied	86 (25.9%)	11 (21.6%)	75 (26.7%)	
Satisfied	146 (44.0%)	28 (54.9%)	118 (42.0%)	
Very satisfied	64 (19.3%)	8 (15.7%)	56 (19.9%)	
Husband requested weight loss	103 (31.0%)	8 (15.7%)	95 (33.8%)	.010*
Chronic diseases	128 (38.6%)	14 (27.5%)	114 (40.6)	0.077
Anxiety	96 (28.9%)	6 (11.8%)	90 (32.0%)	.003**
Depression	129 (38.9%)	4 (7.8%)	125 (44.5%)	<.001**
Total PHQ4 score				
Normal (0-2)	85 (25.6%)	17 (33.3%)	68 (24.2%)	<.001**
Mild (3-5)	150 (45.2%)	32 (62.7%)	118 (42.0%)	
Moderate (6-8)	77 (23.2%)	2 (3.9%)	75 (26.7%)	
Severe (9-12**(**	20 (6.0%)	0 (0.0%)	20 (7.1%)	
Marital satisfaction				
Very dissatisfied	34 (10.2%)	1 (2.0%)	33 (11.7%)	<.001**
Somewhat dissatisfied	46 (13.9%)	1 (2.0%)	45 (16.0%)	
Mixed	114 (34.3%)	16 (31.4%)	98 (34.9%)	
Somewhat satisfied	73 (22.0%)	10 (19.6%)	63 (22.4%)	
Very satisfied	65 (19.6%)	23 (45.1%)	42 (14.9%)	
Frequency of Intercourse				
Less than once/month	34 (10.2%)	0 (0.0%)	34 (12.1%)	<.001**
Once/month	62 (18.7%)	5 (9.8%)	57 (20.3%)	
Once/week	90 (27.1%)	12 (23.5%)	78 (27.8%)	
2-3 times/week	42 (12.7%)	10 (19.6%)	32 (11.4%)	
4-5 times/week	89 (26.8%)	24 (47.1%)	65 (23.1%)	
Daily	15 (4.5%)	0 (0.0%)	15 (5.3%)	
History of sexual harassment/assault	104 (31.3%)	4 (7.8%)	100 (35.6%)	<.001**
Underwent FGC	76 (22.9%)	6 (11.8%)	70 (24.9%)	.040*
Masturbation practice	85 (25.6%)	8 (15.7%)	77 (27.4%)	.078
Husband's sexual dysfunction	118 (35.5%)	3 (5.9%)	115 (40.9%)	<.001**
Think about appearance during intercourse	160 (48.2%)	28 (54.9%)	132 (47.0%)	.297
Believe weight relates to intercourse disorder	98 (29.5%)	9 (17.6%)	89 (31.7%)	.043*

FSFI, female sexual function index; BMI, body mass index; FGC, female genital cutting.

*P*-values were derived from Chi-square tests.

Bold values indicate statistical significance.

* Significant at *P* ≤ 0.05.

** Significant at *P* ≤ 0.01.

Concerning sexual history (Table 3), a considerably higher proportion of women in the dysfunction group reported sexual harassment history and having undergone Female Genital Cutting (35.6% and 24.9% respectively) compared to women in the normal function group (7.8% and 11.8%). Conversely, lower sexual intercourse frequencies were more significantly prevalent among women reporting dysfunction. Participant-reported husband's sexual dysfunction was strongly associated with the participant's own FSFI status (*p* < .001), whereas 40.9% of women with dysfunction reported that their husbands experienced sexual dysfunction. Finally, the belief that the participant's weight relates to an intercourse disorder was significantly associated with FSFI status (*p* = .043). More women in the dysfunction group endorsed this belief (31.7%) compared to women in the normal function group (17.6%). No statistically significant association was found between FSFI status and self-reported masturbation practice (*p* = .078) or whether the participant thinks about their appearance during intercourse (*p* = .297).

The association between BMI and sexual dysfunction categories were illustrated using ORs and their confidence intervals in Table 4. First, the Chi-square test revealed a significant overall association between BMI and sexual dysfunction (*p* = 0.012). The percentage of participants with sexual dysfunction increased with the BMI category: 16.4% in the normal weight group, 22.1% in the overweight group, and 61.6% in the obese group. Second, the univariate logistic regression showed that the chance of sexual dysfunction was significantly higher in the obese group (OR = 2.86, 95% CI: 1.38-5.30, *p* = 0.005) compared to the normal weight group. In contrast, the overweight group showed a non-significant trend towards higher odds of sexual dysfunction (OR = 1.54, 95% CI: 0.684-3.47, *p* = 0.297). These findings strongly suggest that obesity is associated with a higher likelihood of experiencing sexual dysfunction in the population study. Descriptive statistics for the six domains of ArFSFI were summed up in Table 5. The range for each domain varies; desire scores range from 1.2 to 6, while the others range from 0 to 6. The mean scores for each domain were as follows: Desire (3.27), Arousal (3.51), Lubrication (3.89), Orgasm (3.88), Satisfaction (3.69), and Pain (3.55). The total FSFI score, which is a weighted sum of the domain scores, had a mean of 21.78 (SD = 6.36) and ranged from 1.20 to 35.10. Statistically significant lower scores (greater sexual dysfunction) were present in the obese group compared to the normal weight and overweight groups for Lubrication (*p* = 0.002), Orgasm (*p* = 0.014), and the total FSFI score (*p* = 0.029) [(3.69 ± 1.39, Mean Rank = 150.6), (3.73 ± 1.39, Mean Rank = 153.9), and (21.2 ± 5.79, Mean Rank = 154.7) respectively].

**Table 4:** Association between body mass Index (BMI) and sexual dysfunction among married women primary health care attendees in makkah by female sexual function Index Status.

BMI categories	Total	ArFSFI		*P*-value^a^	OR (95% CI)	*P*-value^b^
	*N* = 332	Normal (*n* = 51, 15.4%)	Dysfunction (*n* = 281, 84.6%)			
Normal weight	62 (18.7%)	16 (31.4%)	46 (16.4%)	0.012*	Ref category	
Overweight	76 (22.9%)	14 (27.5%)	62 (22.1%)		1.54 (0.684-3.47)	0.297
Obese	194 (58.4%)	21 (41.2%)	173 (61.6%)		2.86 (1.38-5.30)	0.005**

OR, odds ratio; CI, confidence interval.

*P*-value^a^ is calculated by a Pearson Chi-square test. *P*-value^b^ is calculated by a univariate logistic regression.

Bold values indicate statistical significance.

* Significant at ≤0.05.

** Significant at ≤0.01.

**Table 5:** Comparison of arabic female sexual function Index domains and total scores across BMI categories.

Domain	Range	Factor	Maximum	Normal Weight		Overweight		Obese		Kruskal–Wallis H	*P*-value
				Mean ± SD	Mean rank	Mean ± SD	Mean rank	Mean ± SD	Mean rank		
Desire	1.20-6.00	0.6	3.27 ± 1.16	3.05 ± 1.14	148.4	3.23 ± 1.14	165.6	3.36 ± 1.16	172.7	3.105	0.212
Arousal	0.00-6.00	0.3	3.51 ± 1.36	3.51 ± 1.65	172.1	3.64 ± 1.29	174.3	3.45 ± 1.29	161.6	1.225	0.542
Lubrication	0.00-6.00	0.3	3.89 ± 1.44	4.13 ± 1.64	190.5	4.21 ± 1.31	187.4	3.69 ± 1.39	150.6	12.844	0.002**
Orgasm	0.00-6.00	0.4	3.88 ± 1.45	4.14 ± 1.65	189.8	4.08 ± 1.38	179.7	3.73 ± 1.39	153.9	8.501	0.014*
Satisfaction	0.00-6.00	0.4	3.69 ± 1.54	3.79 ± 1.78	175.8	3.94 ± 1.58	183.0	3.55 ± 1.42	157.1	4.744	0.093
Pain	0.00-6.00	0.4	3.55 ± 1.30	3.74 ± 1.37	179.6	3.66 ± 1.30	173.8	3.44 ± 1.28	159.5	2.664	0.264
FSFI Total	1.20-35.10	–	21.78 ± 6.36	22.3 ± 7.64	181.6	22.7 ± 6.52	184.3	21.2 ± 5.79	154.7	7.087	0.029*

Bold values indicate statistical significance.

* Significant at ≤0.05.

** Significant at ≤0.01.

## 4 Discussion

The present study provides important insights into the complex relationship between BMI and FSD among married women in Makkah, Saudi Arabia. Our findings reveal several key patterns that both align with and diverge from existing literature, while also introducing novel considerations for understanding sexual health in this population. The high prevalence of FSD (84.6%) found in our study population is particularly striking. When comparing our prevalence rates with previous research, several important differences emerge. The 84.6% FSD rate we observed exceeds the 63% prevalence reported by Rouzi et al. (5) among female healthcare providers in Jeddah, 60% among women attending primary care centers in Riyadh reported by Madbouly K et al. (6) and the 68.9% found by Elnashar et al. (19) in Egypt. This is also higher than what was reported by global studies that typically estimate FSD prevalence between 25-63% (3, 13). This discrepancy may be attributed to several factors. The PHC setting of our research may have selected for women with more health concerns than general population studies. Additionally, social and cultural factors specific to Saudi Arabia, including potential stigma around discussing sexual health issues, may have influenced reporting patterns. The use of face-to-face interviews with trained female researchers may have facilitated more honest reporting compared to self-administered questionnaires used in some other studies (2, 12, 19).

The strong association we found between obesity and FSD (OR = 2.86) aligns with studies (9, 10) that reported similar relationships in different populations. The physiological mechanisms underlying this association likely involve multiple pathways. Obesity-related hormonal alterations, particularly in estrogen and testosterone levels, may directly impact sexual desire and arousal (10). Additionally, the vascular changes associated with obesity could impair genital blood flow, affecting lubrication and orgasmic function (23, 24). These biological factors appear to interact with psychological and social dimensions to create a complex web of influences on sexual function. However, our results contrast with the findings of Kadioglu et al. (12) and Sahin et al. (25) who found no significant association between obesity and FSD in a Turkish samples. This discrepancy highlights the importance of cultural context in sexual health research. The different findings may reflect variations in how body weight is perceived and experienced across cultures or differences in study methodologies. Kadioglu et al. study used a different assessment tool for FSD and included a broader age range, which may have contributed to the divergent results (12). Likewise, Sahin et al. study used visceral adiposity index (VAI) instated of BMI to accurately assess obesity and more clearly reflects the metabolic processes that lead to FSD due to the hormonal and inflammatory effects of visceral fat tissue (25) and found no clear relationship was found between FSD and obesity. In addition, in Asci et al. study it was determined that BMI and waist circumference were not good indicators of FSD, while VAI which is a more effective indicator of obesity was associated with the subdomains of FSD (26). These contrasting findings underscore the need for culturally specific research on sexual health issues.

One of the most significant contributions of our study is how partner dynamics influence FSD in obese women. We found that women whose husbands had sexual dysfunction were markedly more likely to report FSD themselves (40.9% vs. 5.9%). This finding supports Thomas et al. (16), who emphasized the crucial role of marital satisfaction and partner health in female sexual function. Our study adds the novel observation that husbands' comments about their wives' weight also significantly impacted sexual function. Women whose husbands had requested weight loss showed higher FSD rates (33.8% vs. 15.7%), suggesting that weight-related stigma within marital relationships may be an important and understudied contributor to sexual dysfunction. This finding has important clinical implications, as it suggests that interventions for FSD in obese women might benefit from including partners in therapeutic approaches.

Our examination of reproductive and gynecological factors yielded several important insights. The association between menstrual irregularities and FSD (*p* = 0.001) may reflect underlying hormonal imbalances that affect both reproductive and sexual function. This finding aligns with a study by Avis et al. (18) on hormonal influences on sexual health across the lifespan. The strong association we observed between reproductive surgeries and FSD supports Addis et al. (14) findings about the sexual consequences of gynecological interventions. The significant relationship between breastfeeding FSD (*p* = 0.004) introduces interesting questions about the role of prolactin and other lactation-related hormones in sexual desire, an area that deserves further investigation (13).

The psychological and lifestyle factors we examined provided additional layers of understanding about FSD in this population. The association between extended sleep duration (>9 h) and higher FSD rates suggests potential links between sleep quality, hormonal regulation, and sexual function that need further exploration. While substance use was relatively rare in our sample, its significant association with FSD (*p* = 0.018) aligns with global data on the negative impact of substance abuse on sexual health (2, 27, 28). The finding of the strong relationship between history of sexual harassment/assault and FSD (35.6% vs. 7.8%), underscore the profound and lasting impact of trauma on sexual wellbeing (13).

Some of our findings contrast with established patterns in the literature, particularly regarding age and education. While many studies report higher FSD prevalence in older women (4, 14), we found FSD across all age groups, with particular peaks in both younger (<30) and older (>45) women. This bimodal distribution may reflect unique cultural pressures in Saudi society, where younger women face expectations about marital sexual performance while older women contend with biological changes and potentially longer-standing marital issues. The education findings were similarly intriguing—contrary to global trends showing higher education associated with lower FSD (3), our university-educated participants had lower dysfunction rates (48.8% vs. 72.5%). This may indicate that education empowers Saudi women to better understand and address sexual health concerns, or it may reflect other socioeconomic factors associated with educational attainment in this context that need further exploration.

Treating patients with FSD by questioning suspected patients through various indicators that may be related to FSD has become a very important issue in clinical practice. Obesity may be one of these indicators. Our results suggest that effective interventions for FSD in obese women should adopt integrated approaches that address both physical and psychological aspects. Weight management programs that incorporate sexual health counseling could be particularly beneficial (29). The strong influence of partner-related factors indicates that couples therapy might be an effective component of treatment. Given the cultural context, developing sensitive, anonymized approaches to sexual health care, possibly including telehealth options, could help overcome barriers to seeking help (30).

Some limitations of our study should be acknowledged. The cross-sectional design prevents us from establishing causal relationships between BMI and FSD. The reliance on self-report measures, introduces potential biases such as social desirability bias, particularly in a cultural context where discussing sexual issues may be stigmatized. Our sample was limited to married women attending PHCs in one city, which may affect generalizability to other populations. The use of BMI as our sole measure of obesity is another limitation. BMI focuses only on weight and does not provide information about the distribution or metabolic effects of fat. Waist circumference, Waist-to-hip ratio, skinfold thickness, visceral adiposity index and body composition analysis could be good alternatives that can more accurately detect obesity and more clearly predict the distribution of fat in the body. Therefore, future studies should include longitudinal studies to clarify causal relationships, investigations of specific hormonal mechanisms linking obesity and FSD, and the development of culturally adapted interventions. The role of inflammatory markers, which are often elevated in obesity and may affect sexual function, represents another promising avenue for research (31). Studies examining the effectiveness of partner-inclusive therapies for FSD in conservative cultural contexts would be particularly valuable.

## 5 Conclusion

Our study makes several important contributions to understanding FSD in obese women within the Saudi context. The high prevalence we observed underscores the need for greater attention to sexual health in this population. The complex interplay between physiological, psychological, and partner-related factors highlights the importance of holistic approaches to assessment and treatment. By integrating sexual health considerations into obesity management and developing culturally sensitive interventions that address the multifaceted nature of FSD, healthcare providers can significantly improve the quality of life for affected women. Our findings also emphasize the need for further research to clarify mechanisms and test interventions in this understudied population.

## Data Availability

The raw data supporting the conclusions of this article will be made available by the authors, without undue reservation.
